# Elucidating the role of diet in maintaining gut health to reduce the risk of obesity, cardiovascular and other age-related inflammatory diseases: recent challenges and future recommendations

**DOI:** 10.1080/19490976.2023.2297864

**Published:** 2024-01-04

**Authors:** Tariq Aziz, Nageen Hussain, Zunaira Hameed, Lin Lin

**Affiliations:** a School of Food & Biological Engineering, Jiangsu University, Zhenjiang, China; b Institute of Microbiology and Molecular Genetics, New Campus, University of the Punjab, Punjab, Lahore

**Keywords:** Inflammatory diseases, microbiota, obesity, probiotics, polyphenols

## Abstract

A healthy balanced diet is crucial in protecting the immune system against infections and diseases. Poor diets, such as the Western diet, contribute to the development of metabolic diseases, hypertension, and obesity. Microbiota, primarily composed of different microorganisms and residing in the gastrointestinal tract (GIT), also play a significant role in maintaining gut health. Polyphenols and probiotics found in fruits, vegetables, whole grains, legumes, nuts, and seeds promote gut health and support the growth of beneficial bacteria. Different types of diets, their categories, and their impact on health are also mentioned. The relationship between diet, gut health, and the risk of developing obesity, cardiovascular diseases, and inflammatory diseases is discussed in this review article. The rationale behind the review concludes future recommendations for maintaining gut health and reducing the occurrence of obesity, cardiometabolic diseases, and other inflammatory diseases. There is also the need for standardized research methods, long-term studies, and translating scientific knowledge into practical dietary recommendations.

## 1. Introduction

To protect the human body from certain infections and diseases, the immune system plays a vital role. A healthy and balanced diet can help support immune function by providing essential nutrients and reducing inflammation.^1^ However, the relationship between diet and immune function is complex because genetic, immunological and environmental factors (lifestyle, infections, and hormonal imbalance) are involved that can also affect immune health.^2^ Intestinal health plays a crucial role in the digestion and absorption of nutrients and elimination of waste from the body. Intestinal health refers to the well-being and proper functioning of the intestines, which are part of the digestive system.^3^ Maintaining a healthy intestinal tract is important for the overall health and well-being of the small and large intestines. One key factor contributing to intestinal health is that a nutritious and balanced diet is essential for good intestinal health as it promotes regular bowel movement. Probiotics are key to maintaining intestinal health.^4^ Diet is a very important factor in human life which has a direct influence on host physiology as well as indirect effects arbitrated by microbiota and its metabolome. Diet, the gut microbiota, and the host health interact in a complicated manner.^5^ The diet which may be linked with the host’s health is the one that has high fiber content, unsaturated fatty acids, and polyphenols and is lower in saturated fats, sodium content and processed carbohydrates. Depending on the diet, long-term dietary impacts that directly affect the host physiology can either be beneficial or harmful. For instance, a diet high in fiber and unsaturated fatty acids, such as the Mediterranean diet, is linked with a decreased risk of metabolic syndrome and cardiovascular disease (CVD).^6,7,56^ Conversely, a diet high in refined carbohydrates, saturated fats, and low in fiber, known as the Western diet, is linked with an increased risk of obesity and CVD.^8,9^ Additionally, many researchers have explained and shown how certain diets can be used to treat a variety of diseases, such as how a gluten-free diet is the first line of treatment for celiac disease^10^ and how exclusive enteral nutrition (EEN) is a successful treatment for Crohn’s disease.^11,12^ The rationale behind conducting the review enlightens the role of diet and its impact on reducing the risk of obesity, cardiovascular and various inflammatory diseases.

**Figure 1.**
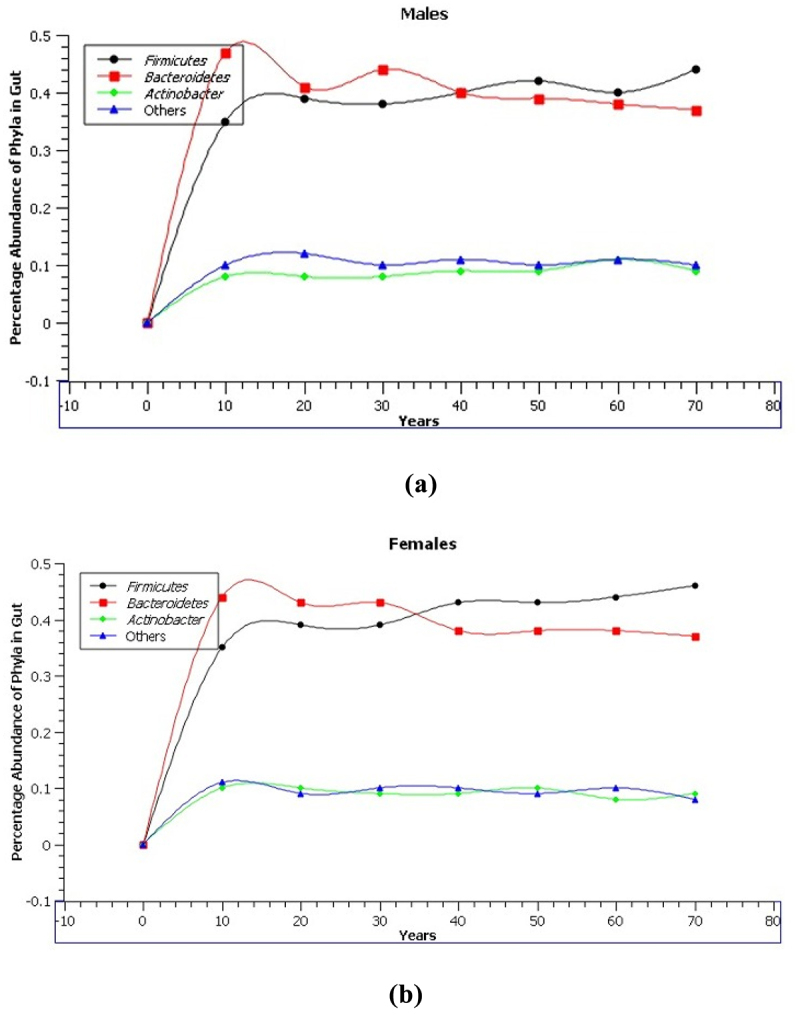
Percentage abundance of phyla present in gut according to age groups (A) males (B) females.

More specifically, inadequate diets lead to the onset of metabolic disorders such as obesity, hypertension, and cardiovascular issues.^13^ As a result, nutrition is also associated with the development of many diseases by triggering inflammation, which is tightly controlled and involves the production of inflammatory mediators like cytokines and chemokines by both immune and non-immune cells with specialized functions in response to pathogens or tissue damage. Leucocytes are afterward dispatched to the infection site and eliminate the infection, repair tissue damage, and finally guarantee the remission of inflammation.^14^ On the other hand, a state of persistent inflammation with the resulting disease may emerge if the initial inflammatory stimulation cannot be eliminated. An inadequate inflammatory response is now understood to contribute to a wide range of metabolic and immunological disorders.^15^ In Western countries, dietary patterns have changed in recent decades along with the increased consumption of highly processed foods with high caloric density, low fiber content and high content of processed carbohydrates, saturated fats, and salt leading to various metabolic diseases such as obesity, cardiovascular diseases, and metabolic syndrome.^16^ A healthy gut barrier prevents the entry of harmful substances into the bloodstream, reducing the risk of inflammation and immune system activation. The gut microbial flora is an environmental element that interacts with nutrition and influences health outcomes, some of which involve metabolites that are produced by the gut flora from dietary elements and can have an impact on the host. The human microbiota is made up of bacteria, viruses, fungi, archaea and protozoans that can be found in the skin, mouth, vagina, GIT and respiratory system.^17^ The gastrointestinal tract (GIT) colonizes around 70% of the microbiota and contains more than 100 trillion bacteria.^18^ *Firmicutes* and its surrounding genera, *Lactobacillus, Bacillus, Clostridium, Enterococcus, Ruminococcus, Eubacterium, Faecalibacterium*, and *Roseburia* are the principal phyla present in the gut microbiota. Bacteroidetes is the second phylum, which includes *Bacteroides* and *Prevotella* and is followed in descending order by the phyla *Acinetobacteria* and others. The percentage abundances of these phyla according to different age groups and gender are given in Figure 1.^19^ However, according to one research, the relative abundance of dominant gut bacterial phyla, *Firmicutes* and *Bacteroides*, didn’t change between the participants following the Western and Mediterranean diets.^20^ It is well established that the proportion of gut microbes at the phylum level differs depending on various factors including age, race, dietary patterns etc. For example, adults with dietary patterns rich in plant-based foods have a higher abundance of *Firmicutes* than *Bacteroides*. On the other hand, keep in mind that children and old people have weak immune systems and thus weak gut flora as compared to teenagers and young adults but it also depends on the link between dietary patterns and age distribution.^21^

### 1.1. Types of diet

There are different types of diets, and their categorization depends upon the intake of specific groups of foods in different parts of the world.^22^

#### 1.1.1. Mediterranean diet

The Mediterranean diet and gut microbiota have gained great attention in the last few years because of their significant health benefits. The traditional eating practices of the Mediterranean Sea adjacent to nations like Italy, Greece, and Spain served as the inspiration for the diet known as the Mediterranean diet.^23^ While restricting the consumption of processed food items, red meat, and sweets, it emphasizes the consumption of entire foods, such as veggies, legumes, whole grains, fruits, nuts, seeds, seafood, and olive oil.^24^ One of the fascinating aspects of the Mediterranean diet is its positive impact on the microbiota of the gut. The significant community of bacteria that live in our GIT is referred to as the gut microbiota. These microbes are essential for digestion, nutritional absorption, immune system functioning, and general well-being. According to studies, the Mediterranean diet lowers the prevalence of dangerous bacteria while encouraging the growth of helpful gut flora.^25^ The high intake of fiber-rich diets such as fruits, legumes, and vegetables, provides a favorable environment for the growth of beneficial bacteria. These fibres are known as prebiotics, which serve as nourishment for the gut microbiota. By consuming prebiotic-rich foods, individuals following the Mediterranean diet can support the growth of bacteria like *Bifidobacteria* and *Lactobacillus*, which have been associated with various benefits of health i.e., improved function of the immune system and reduced inflammatory conditions.^26^ Furthermore, the Mediterranean diet is abundant in polyphenols which are plant compounds obtained from fruits, vegetables, and olive oil. These have antimicrobial properties, influencing the diversity of the gut microbiota, and are antioxidants. According to studies, polyphenols can promote a healthier microbiome by increasing the number of helpful bacteria and reducing the growth of harmful bacteria in the gut. Consuming oily fish, which is a cornerstone of the Mediterranean diet and includes salmon and sardines, also helps to maintain a balanced gut flora. Omega-3 fatty acids, which have anti-inflammatory qualities, are abundant in fish species with fat in soft tissues around the gut. These fatty acids can alter the composition of the gut microbiota, encouraging the growth of helpful bacteria and lowering gut inflammation.^27^ Overall, the Mediterranean diet provides a variety of components that support diverse and beneficial gut microbiota. By following this dietary pattern, individuals can potentially improve their gut health, enhance nutrient absorption, and lower the occurrence of various ailments, including type 2 diabetes, obesity, and cardiovascular diseases.^28^ Factors such as genetics, lifestyle, and existing gut microbiota composition can influence the outcomes. However, the Mediterranean diet’s emphasis on whole, unprocessed foods, rich in fiber, polyphenols, and healthy fats, provides a solid foundation for nurturing a healthy gut microbiota and overall well-being.^29^

#### 1.1.2. Dietary approaches to stop hypertension (DASH) diet

While limiting sodium intake to lower blood pressure, the DASH diet emphasizes a diet comprised of vegetables, fruits, whole grains, lean meats, and low-fat dairy products.^30^ The high intake of fiber-rich foods in the DASH diet, such as fruits, whole grains, and vegetables can provide nourishment for beneficial gut bacteria. Such prebiotics encourage the development and activity of probiotic bacteria like *Lactobacillus* and *Bifidobacteria*. The acids (SCFAs) which are produced by these bacteria give energy to the intestinal cells, improve the function of the intestinal barrier, and also have anti-inflammatory properties.^31^ The DASH diet also promotes the use of low-fat dairy items because they include probiotics. Probiotics are living yeast or bacteria that when taken have positive effects on health. They can encourage the development of good bacteria and aid in gut microbiota balance restoration. High-sodium diets have also been linked with negative alterations in the gut microbiota, including reduced diversity of the strains. By reducing sodium intake and increasing the consumption of whole foods, the DASH diet may create a more favorable environment for a diverse and beneficial gut microbiota.^32^

Studies have demonstrated the potential positive effects of the DASH diet on gut microbiota, further research is needed to fully understand the specific mechanisms and long-term impacts. Factors such as individual variations in gut microbiota, genetic factors, and overall dietary habits can influence the outcomes. The DASH diet, with its emphasis on whole foods, fiber-rich fruits and vegetables, low-fat dairy products, and limited sodium intake, may contribute to a healthier gut microbiota.^33^ By supporting the growth of beneficial bacteria and promoting SCFA production, the DASH diet may improve gut health, enhance nutrient absorption, and potentially reduce the risk of various diseases.^34^ Incorporating the DASH diet into a healthy lifestyle can have a positive impact on both blood pressure control and gut microbiota composition, leading to overall improved health.^35^

#### 1.1.3. Vegan diet

The vegan diet and gut microbiota have gained considerable attention in recent years due to their potential impact on health. A vegan diet is a plant-based dietary pattern that excludes all animal-derived products, including meat, dairy, eggs, and honey, and focuses on consuming fruits, vegetables, whole grains, legumes, nuts, and seeds. This dietary approach has been associated with numerous health benefits, and emerging research suggests that it may also influence gut microbiota. Several studies have examined the relationship between a vegan diet and gut microbiota composition. Research indicates that individuals following a vegan diet tend to have a distinct microbial profile compared to those consuming an omnivorous diet.^36^ Vegan diets are typically high in fiber, which serves as a prebiotic for gut bacteria. Increased fiber intake from plant-based foods can stimulate the growth of beneficial bacteria like *Bifidobacterium* and *Prevotella*, which are associated with improved gut health and enhanced immune function.^37^ Moreover, vegan diets are often rich in phytochemicals, which are plant compounds with various health benefits. Phytochemicals can act as antioxidants and possess antimicrobial properties, influencing the composition and activity of gut microbiota. Studies have shown that plant-based diets, can increase the abundance of certain beneficial bacteria and decrease the levels of potentially harmful bacteria in the gut.^38^ Additionally, the absence of animal-derived products in a vegan diet may alter the production of certain metabolites in the gut, such as SCFAs. SCFAs, produced by the fermentation of dietary fiber by gut bacteria, play a vital role in gut health and overall well-being. Vegan diets, rich in fiber, have been associated with increased SCFA production, particularly butyrate, which has anti-inflammatory properties and supports the health of intestinal cells.^39^ Thus, a vegan diet has been associated with unique changes in gut microbiota composition. The high fiber content and rich phytochemical profile of a vegan diet contribute to a favorable gut environment that supports the growth of beneficial bacteria and the production of important metabolites. However, further research is needed to fully understand the complex interactions between a vegan diet, gut microbiota, and overall health.

#### 1.1.4. Western diet

**Figure 2.**
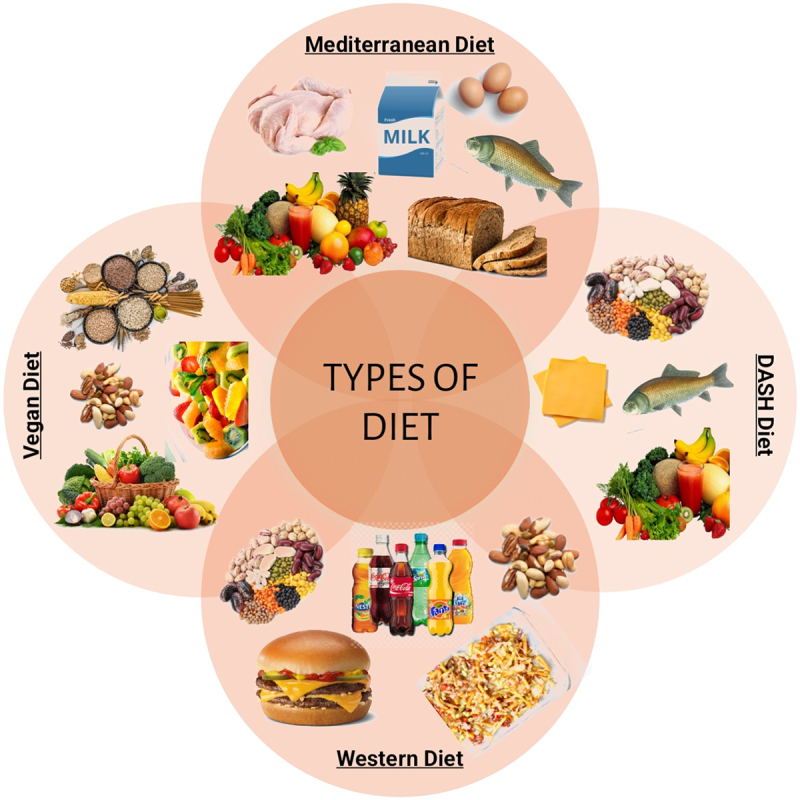
Different types of diet.

A prevalent dietary pattern in industrialized nations, notably in North America, Europe, and Australia, is the Western diet, sometimes known as the Western pattern diet. A high diet of red and processed meats, sweetened meals and drinks, refined cereals, high-fat dairy products, and foods with added sugars and fats are some of its defining characteristics.^40^ On the other hand, fruits, vegetables, whole grains, and legumes are typically in short supply in the Western diet. A higher risk of certain chronic illnesses, such as obesity, type 2 diabetes, cardiovascular disease, several kinds of cancer, and metabolic syndrome has been linked to the Western diet. Its high intake of foods that are high in calories but low in nutrients, as well as its low fiber content and excessive consumption of added sugars and harmful fats, all have a detrimental impact on health.^41^ Figure 2 highlights some of these types of diets.

### 1.2. Gut microbiota

**Figure 3.**
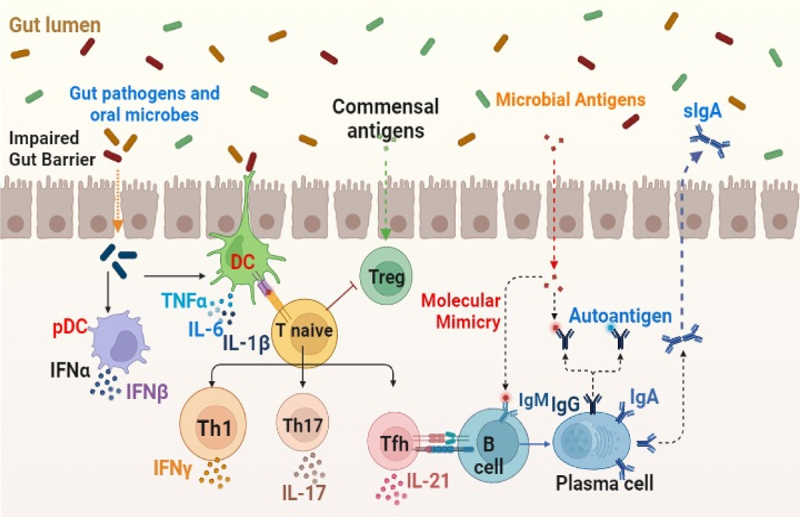
Role of gut microbiota in autoimmune diseases.

The beneficial bacteria that support the integrity of the gut barrier and control the activity of the immune system in the gut are *Faecalibacterium prausnitzii*, *Akkermansia muciniphila*, and *Bifidobacterium*. A kind of helpful bacteria called *bifidobacteria* has several advantages on a person’s health including strengthening the immune system, reducing inflammation, and encouraging good digestion.^42^ A common type of bacteria in the stomach called *Lactobacillus* has been shown to have a beneficial impact on gut health. As a result, it has been connected to improve the function of the intestinal barrier, reducing inflammation, and boosting overall good digestive health.^43^ IBD may be prevented and treated with the help of the bacterium *Faecalibacterium prausnitzii*, which has anti-inflammatory capabilities.^44^ The species of bacteria known as *Akkermansia muciniphila* is regarded as being essential for preserving gut health. Additionally, it has been associated with a decrease in inflammation, better gut barrier performance, and defense against obesity and metabolic illnesses.^45^ *Prevotella*, *Bacteroides*, and *Ruminococcus sp*., on the other hand, create inflammation and contribute to the development of various diseases, including some autoimmune disorders, by disrupting the functioning, as illustrated in Figure 3.^46^

**Figure 4.**
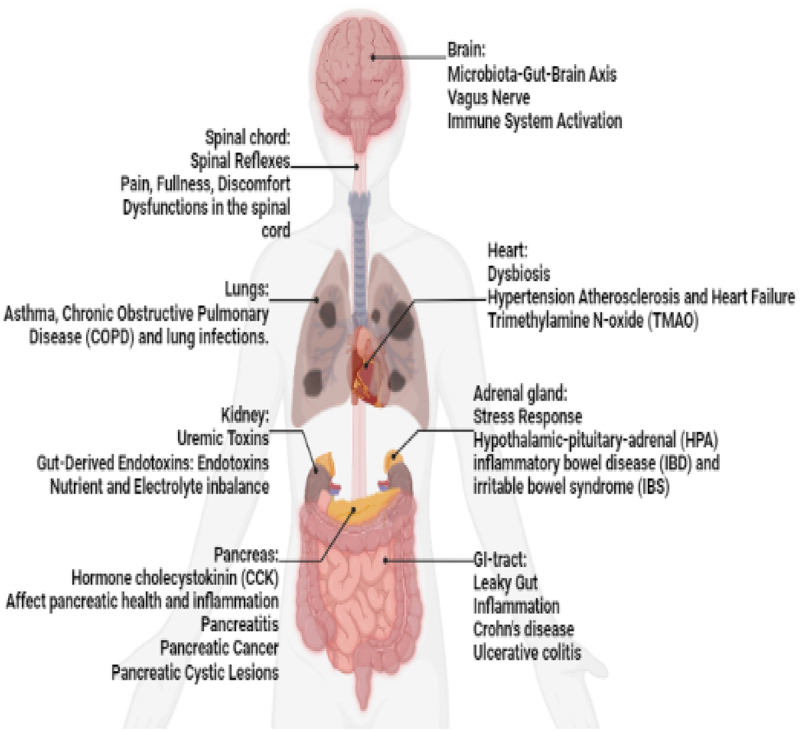
The human gut-brain axis.

The bacterial composition changes throughout a person’s life and is influenced by genetics, age, how quickly bacteria pass through the gut, and a variety of environmental variables, such as the delivery method, breastfeeding, the microbiota of the mother, nutrition, lifestyle, medicine, and overall health.^47^ Indeed, environmental influences on the gut microbiota seem to be even more significant than genetic influences, and recent research indicated that up to 20% of the diversity in microbiota across individuals was related to food, medicine, and body composition. Furthermore, it has been demonstrated that diseases like obesity and cardiometabolic diseases are diet-dependent and therefore, the intake of fruits, vegetables and low-fat diet is healthy.^48^ The microbiota of the gut is also linked with the brain and its functioning. The gut-brain axis is a well-known concept, and their relationship is discussed in this review article. The microbiota-gut-brain axis refers to the mutually beneficial interaction between the gut bacteria, the gut, and the brain. The gut microbiota affects the brain and behavior in several ways, including neurotransmitter synthesis, inflammation control, and immune system modulation. Stress, medications, and nutrition all have an important effect on the microbiota of the gut, and imbalances in this population have been linked to mental health problems.^49^ Figure 4 depicts how the gut-brain axis is connected.

The vagus nerve, a lengthy cranial nerve, connects the brain to numerous organs, including the gut, and is instrumental in the bidirectional communication between the gut and the brain. It relays information about gut health, inflammation, and satiety to the brain by transmitting signals from the gut. Additionally, the vagus nerve regulates gut motility, immune responses, and secretion.^50^ The gut is a crucial site for immune system activation, with the gut-associated lymphoid tissue (GALT) housing a multitude of immune cells that function in protecting against pathogens and maintaining immune homeostasis. Immune dysregulation in the gut, such as increased inflammation or decreased immune responses, might have systemic implications and contribute to a variety of inflammatory illnesses.^51^ Dysbiosis, defined as an imbalance or disruption in the diversity of the gut microbial flora, can occur as a result of a variety of circumstances including poor diet, stress, antibiotics, or certain diseases.^52^ It has been linked to increased inflammation, altered immunological responses, and an increased chance of acquiring illnesses like metabolic disorders, obesity, and inflammatory bowel disease. Dysbiosis in the gut microbiota has been associated with the development of hypertension, atherosclerosis, and heart failure. The gut microbiota can create compounds that regulate blood pressure, cholesterol metabolism, and systemic inflammation, all of which contribute to cardiovascular health.^53^ Trimethylamine N-oxide (TMAO) metabolite, which is formed by gut bacteria from dietary components such as choline and carnitine, has been linked to an elevated risk of cardiovascular disease. TMAO can cause inflammation, alter cholesterol metabolism, and impair platelet function, all of which can lead to cardiovascular problems. Through the stomach, the Hypothalamic-Pituitary-Adrenal (HPA) axis affects the stress response. A dysbiosis of the intestinal tract and enhanced gut permeability can have an impact on how the HPA axis responds to stress and how it is modulated.^54^ Chronic gastrointestinal tract inflammation is a hallmark of IBD, which involves ulcerative colitis and Crohn’s disease. Pathogenesis is influenced by gut dysbiosis, gut barrier failure, and immune system activation. Irritable bowel syndrome (IBS) is a functional gastrointestinal illness characterized by stomach pain, bloating, and changes in bowel habits that may be influenced by gut-brain connections, changes in the gut microbiota, and low-grade inflammation.^5^ A leaky gut, defined by increased intestinal permeability, can contribute to a variety of health problems by causing systemic inflammation caused by chemicals flowing through the gut lining into the bloodstream.^55^

### 1.3. Diet with polyphenols and their impact on gut health

**Figure 5.**
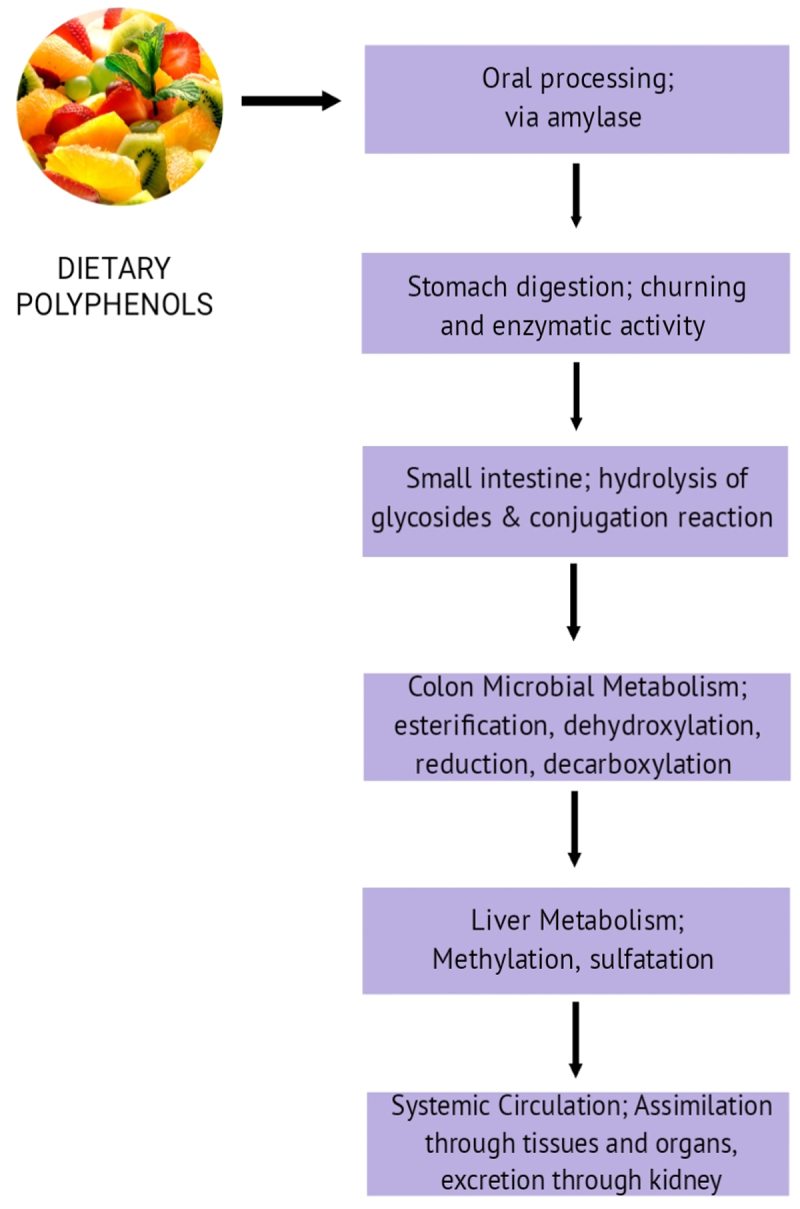
Metabolism of polyphenols by gut microbiota.

Polyphenols are known for their antioxidant properties therefore consuming foods that are naturally high in polyphenols, such as fruits, whole grains, vegetables, legumes, seeds, and nuts are emphasized in diets.^56^ These foods include both flavonoids (such as anthocyanins, flavanols, and flavones) and non-flavonoid polyphenols (such as stilbenes, and lignans), which include a wide variety of polyphenols. In addition to having anti-inflammatory and antioxidant properties, polyphenols have been linked to several health advantages, including lowering the chance of developing chronic illnesses including cardiovascular disease, certain malignancies, and neurological disorders.^57^ A typical adult consumes up to 1 g of dietary polyphenols each day, which is up to 100 times more than they consume in terms of carotenoids, and vitamin E, and roughly 10 times more than they consume in terms of vitamin C. The majority of dietary polyphenols are processed by the gut microbiota in the large intestinal tract, where they accumulate as opposed to being assimilated in the small intestinal tract. Therefore, gut bacteria are necessary for the transformation of the natural polyphenolic compounds into low-molecular-weight products that can be readily absorbed and enhance the health of the host.^58^ The metabolism is shown in the form of a flowchart (Figure 5).

Dietary polyphenols may alter the gut microbiota of the host, which may have an impact on the host’s metabolism. Additionally, the intestinal microbiota can convert polyphenols into bioactive, low-molecular-weight phenolic compounds, which modifies the regulatory and metabolic network.^52^ The composition and functionality of the gut microbiota are greatly influenced by dietary polyphenols. These polyphenols encourage the growth of healthy microorganisms that are favorable for human health, including the well-known probiotics *Lactobacillus* and *Bifidobacterium*. To name a few benefits of their presence, they lower lactose intolerance, minimize diarrhea and constipation, improve irritable bowel syndrome symptoms, and prevent inflammatory bowel disease.^59^ Additionally, *Clostridium histolyticum* and *Clostridium perfringens* can’t grow well due to polyphenols. Dietary polyphenols can influence the *Firmicutes* to *Bacteroides* ratio, which has been linked to body weight and is higher in obese individuals. Four specific dietary polyphenols – rutin, quercetin, chlorogenic acid, and caffeic acid – have been demonstrated to be able to lower the *Firmicutes*-to-*Bacteroides* ratio in *vitro* studies on the gut microbiota.^60^

Dietary polyphenols, notably flavanols and flavones in *Staphylococcus*, disrupt bacterial cell membranes and increase membrane permeability. Gram-positive bacteria are more sensitive to them while Gram-negative bacteria are more resistant to their antibacterial activities. However, exceptions are there, Gram-negative *Salmonella* and *Escherichia coli* are prevented from growing by ingested polyphenols, whereas Gram-positive lactic acid bacteria are not. Membrane function is disturbed by contact with bacterial cell surfaces, which prevents bacterial growth. Tea polyphenols, such as catechins, attach to cell membranes and have antibacterial and anticancer properties as a result. *Staphylococcus* cell membranes are damaged by EGCG, which also inhibits the growth of biofilms and neutralizes enterotoxin B. Polyphenols influence metabolic health by altering the bacterial cell membranes.^61^ The summarized Table 1 is given below regarding polyphenols, their natural sources and how they affect microbial flora of the gut.

**Table 1.** Polyphenols, its natural sources and impact on microbiota.

Polyphenol	Source	Impact	Reference
Anthocyanins	Blackcurrant, Tart cherry	Increase in *Lactobacillus, Bifidobacterium*; Decrease in *Bacteroides* spp., *Clostridium* spp.	^62^
Polyphenols, phenolic acids	Apple	Increase in *Lactobacilli, Streptococcus sp*; Decrease in lecithinase-positive *Clostridium, Pseudomonas, and Enterobacteriaceae*	^55^
Flavanols	Cocoa	Increase in *Bifidobacterium, Lactobacillus*, E. rectale*-C. coccoides*; Decrease in *Clostridium*; In low–cocoa group, increase in *Clostridium*, E. rectale*-C. Coccoides*	^60^
Proanthocyanins, Apigenin	Blueberry	Increase in *Bifidobacteria*, spp. Of *Prevotella*, *Bacteroides* spp., and *C. coccoides*; Decrease in the species of *Enterococcus.*	^63^
Flavonoid, Isoflavone	Almond, Orange	Increase in *Bifidobacterium* spp. and *Lactobacillus* spp.; Decrease in pathogen *C. perfringens, C. leptum, and B. coccoides.*	^64^
Proanthocyanidins	Cranberry	Increase in *Bacteroidetes*, *Lachnospira* and *Anaerostipes*.; Decrease in *Clostridia, Firmicutes, and Oribacterium*	^65^
Catechins, Quercetin	Green tea	Increase in *Firmicutes, Actinobacterium*. Decrease in *Bacteroidetes*	^66^
Cocoa flavanols	Dark chocolate	Increase in *Lactobacillus*; Decrease in *Bacteroidetes*	^67^

### 1.4. Diet with probiotics and their impact on gut health

**Figure 6.**
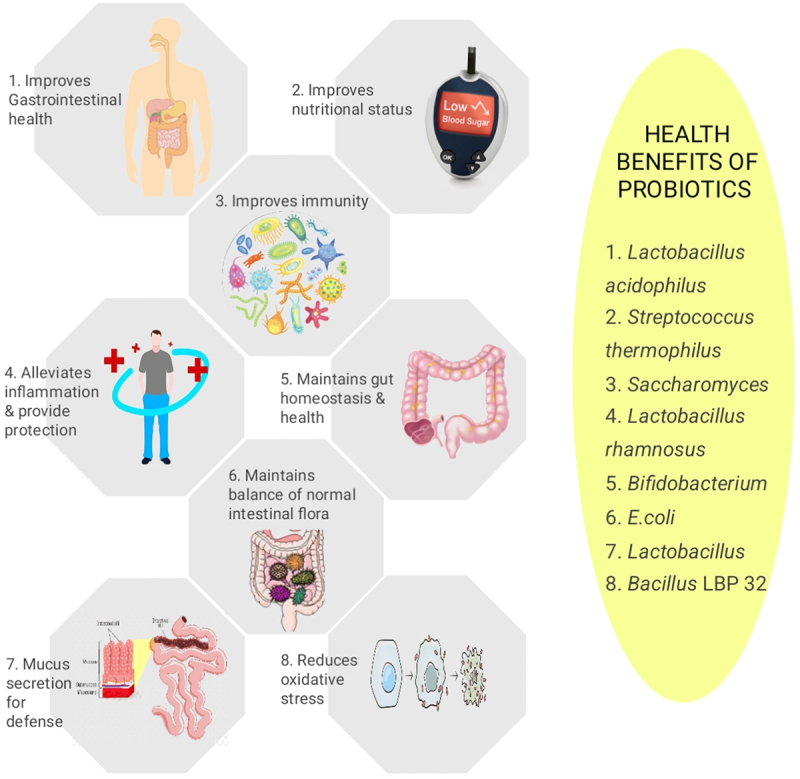
Benefits of probiotics on the overall health of an individual.

Probiotics on the other hand, when taken in higher amounts, induce several health benefits to the host. They improve digestion, boost immunological function, maintain an appropriate equilibrium of gut bacteria, and may also have additional advantageous impacts on general health, as illustrated in Figure 6. A probiotic-rich diet includes fermented foods that naturally contain live beneficial bacteria. Examples of foods that are rich in probiotics include kefir, yogurt, sauerkraut, tempeh, kimchi, and certain types of cheese.^68,69^ These foods undergo fermentation processes that promote the growth of specific strains of beneficial bacteria. Probiotic supplements are also available, providing concentrated amounts of specific strains of bacteria. These probiotics are categorized as dairy and non-dairy probiotics. Consuming dairy probiotics can have a positive impact on the microbiota of the gut.^70^ These probiotics can help restore and maintain a healthy balance of microorganisms in the gut. They can increase the amount of good and healthy bacteria and decrease the growth of certain harmful bacteria. Dairy probiotics commonly belong to the bacterial group which produces lactic acid and have *Lactobacillus* and *Bifidobacterium* species .^71^ Several studies have suggested that changes in the gut microbiota composition and diversity can influence the development and progression of various diseases. Imbalances or disruptions in the gut microbiota, known as dysbiosis, have been associated with conditions like IBD, IBS, obesity, diabetes, allergies, and even mental health disorders. Dairy probiotics can potentially modulate the gut microbiota and help prevent or alleviate certain diseases. They can enhance the production of short-chain fatty acids, vitamins, and other metabolites that promote intestinal health.^72^ Probiotics may also improve gut barrier function, strengthen the immune response, and reduce inflammation in the gut. However, it’s important to note that the effects of dairy probiotics on gut microbiota and disease prevention or treatment can vary depending on the specific strains used, the dosage, and individual factors.^73,74^

Not all probiotics have the same benefits, and their mechanisms of action can differ. Therefore, it’s crucial to choose probiotic products that have been scientifically studied and shown to have beneficial effects on the desired health outcomes. Some common dairy probiotics are *Lactobacillus acidophilus, Bifidobacterium lactis, Lactobacillus casei, Streptococcus thermophilus*, and *Lactobacillus rhamnosus*. *Lactobacillus* shows antirotaviral and antibacterial activity by promoting metabolites like bacteriocin, non-bacteriocin and lactic acid. Thus they promote a healthy balance of gut bacteria and improve lactose digestion i.e., alleviate symptoms of lactose tolerance and support digestive health. *L. rhamnosus* has been studied for its potential benefits in supporting the immune system and promoting gastrointestinal health.^75^ Several gut diseases occur due to microbial alteration e.g., IBD, IBS, hepatic encephalopathy, obesity, autoimmune illnesses, mental problems, and acute infectious diarrhea. Due to increased microbial diversity, probiotics have been useful for IBD patients.^76^

Non-dairy probiotics are probiotic supplements or foods that do not contain dairy products.^68,77–80^ They are commonly found in fermented foods like yogurt, kefir, and sauerkraut, which are traditionally dairy-based. However, due to various dietary restrictions, allergies, or personal preferences, non-dairy probiotics have gained popularity as an alternative for individuals who cannot or choose not to consume dairy products.^72,81–84^ Non-dairy probiotics can positively impact the gut microbiota by introducing beneficial live microorganisms.^85–91^ When consumed, these probiotics can help restore or maintain a healthy balance of gut bacteria. The introduction of these beneficial bacteria can help suppress the growth of harmful microorganisms and promote a diverse and stable microbial community in the gut. Studies have shown that alterations in the gut microbiota, often referred to as dysbiosis, have been associated with several diseases and conditions, including gastrointestinal disorders, metabolic disorders, autoimmune diseases, and even mental health disorders.^92–96^ By influencing the gut microbiota, non-dairy probiotics may offer potential health benefits and contribute to the prevention or management of these conditions. Some of these non-dairy probiotics with their sources are given in Table 2.

**Table 2.** Sources of non-dairy probiotics.

Non-dairy probiotics	Sources of Non-dairy probiotics
Fermented Veggies	sauerkraut, pickles, and kimchi
Fermented Soy Products	soy products like tempeh and miso
Kefir	Non-dairy kefir
Probiotic Supplements	capsules, tablets, and powders

Following are a few examples of how non-dairy probiotics may impact specific diseases:^97^

1. Gastrointestinal Disorders: It has been investigated whether non-dairy probiotics can help treat antibiotic-associated diseases such as IBD, IBS, and diarrhea. They can lessen inflammation, restore the proper balance of bacteria in the gut, and treat the symptoms brought on by these conditions.
2. Metabolic Disorders: Some studies suggest that certain strains of non-dairy probiotics may have a positive impact on metabolic health. They may help improve glucose metabolism, reduce insulin resistance, and influence lipid metabolism, potentially benefiting individuals with conditions like obesity and type 2 diabetes.
3. Immune Function: The gut microbiota plays a critical role in modulating the immune system. Non-dairy probiotics may enhance immune function by stimulating the production of beneficial compounds, supporting the development of immune cells, and promoting a balanced immune response. This could be beneficial for conditions such as allergies, autoimmune diseases, and respiratory infections.
4. Mental Health: Emerging research suggests a link between the microbiota of the gut and mental problems. Non-dairy probiotics may have a role in improving symptoms of depression, anxiety, and stress by influencing the gut-brain axis, a bidirectional communication pathway between the gut and the brain.

It’s important to note that the effects of non-dairy probiotics on gut microbiota and diseases can vary depending on the specific strains and formulations used, as well as individual factors. Also, use of proper probiotics is recommended.

#### 1.4.1. Mechanism of action of probiotics

**Figure 7.**
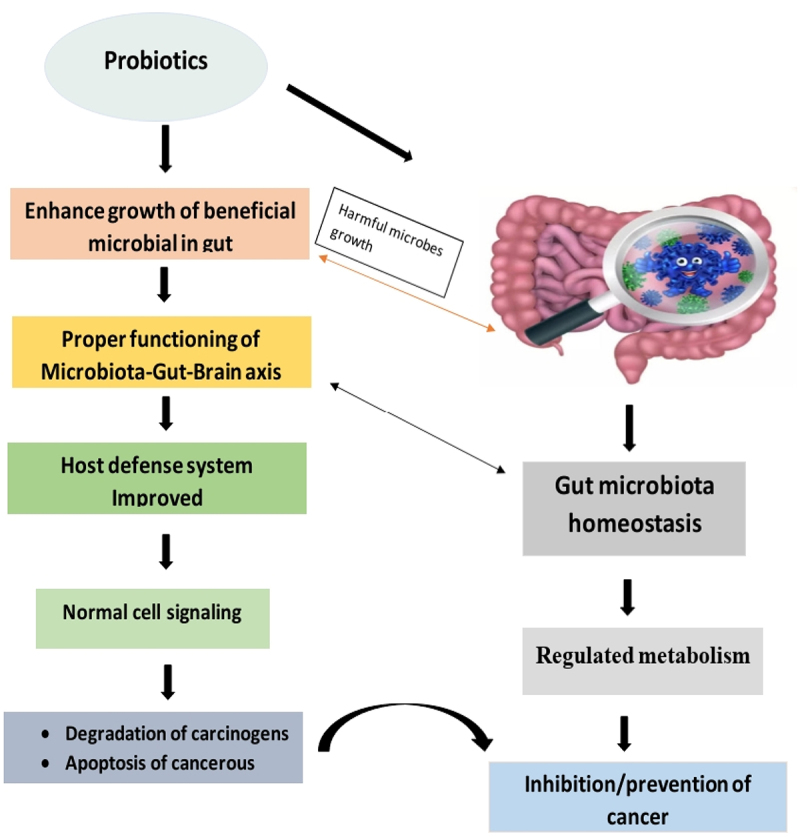
Mechanism of action of probiotics.

*Lactobacillus* species produce lactic acid and help create an acidic environment leading to the inhibition of the growth of harmful bacteria. Apart from that, they are involved in the competitive exclusion of pathogenic bacteria by occupying the sites of adhesion and nutrient intake. These bacteria promote mucin production and regulate the production of tight junction proteins, preventing the entry of toxins and pathogens.^98^ Other bacteria including strains of *Streptococcus* and *Enterococcus*, can ferment dietary fibres, producing short-chain fatty acids and thus helping in maintaining the health of gut epithelium. These can also interact with immune cells by influencing the production of cytokines which regulate immune response. Some *Enterococcus* sp. possess bile salt hydrolase activity, allowing them to metabolize and modify bile salts. This can have implications for bile acid homeostasis and overall gut health.^99^ The summarized mechanism of action of these probiotics is described in Figure 7 given below.

### 1.5. Diet with prebiotics and their impact on gut health

Prebiotics are the compounds that speed up the growth and activity of beneficial bacteria in the gastrointestinal tract. These are present in foods like yogurt, whole grains, bananas etc. Prebiotics have the potential to improve gut health balancing the gut microbiota. These prebiotics are fermented by the bacteria present in the gut and produce short-chain fatty acids, e.g., propionate, butyrate, and acetate. These short-chain fatty acids provide membrane integrity of the intestine and absorption of minerals, lower body weight, improve immunity, and modulate the cardiometabolic and inflammatory biomarkers. Also, the intake of prebiotics favors the growth of beneficial bacteria, such as *Lactobacillus* and *Bifidobacterium*, which are responsible for inhibiting the growth of harmful bacteria.^100^

### 1.6. Obesity, cardiovascular, aging and other inflammatory diseases

#### 1.6.1. Obesity and cardiometabolic diseases

A high-fat, high-sugar diet, such as the Western diet, has been linked to an elevated risk of obesity. Recent studies have linked obesity to the microbial flora in the stomach. Western diet has negative impacts on the variety and composition of the gut microbiota. According to studies, those who follow a Western diet have a less diversified gut microbiome that is marked by having a larger number of hazardous bacterial species and a lesser number of helpful bacterial species. Increased energy absorption from food, inflammation, and metabolic dysfunction are all linked to this changed microbial makeup and can lead to weight gain and obesity.^101^ In addition, the gut microbiota is essential for controlling metabolism, fat storage, and energy balance. The creation of short-chain fatty acids (SCFAs), the extraction of calories from food, and the modulation of hormones involved in hunger control are all influenced by the microbial makeup. These procedures can be hampered by dysbiosis, or an imbalance in the gut microbial ecology, which can also encourage weight gain. The gut flora is crucial to maintaining gut health as well as general well-being as it helps in digestion, nutritional absorption, immune function, synthesis of a few vitamins, SCFAs, and nutrient absorption. IBD, obesity, and abnormalities in metabolic function have all been associated with dysbiosis or imbalances in the composition of the gut microbiota. Changes in the composition of the microbes in the gut, which interacts with the host’s immune system, may affect immunological function. SFAs, particularly those derived from animal-based products, can exacerbate inflammation and boost the body’s immune response, which may contribute to developing inflammatory diseases. Some PUFAs, such as omega-3 fatty acids, on the other hand, have anti-inflammatory properties and may help to favorably modulate immune responses. Dysbiosis and inflammation caused by a poor diet high in SFAs and low in BDFAs may have an impact on the onset of many diseases. For instance, differences in the gut microbiota’s makeup and activity have been linked to metabolic disorders and obesity, and dysbiosis has been linked to the etiology of IBD. Dysbiosis-induced inflammation may have an impact on cardiovascular health.^102^

**Figure 8.**
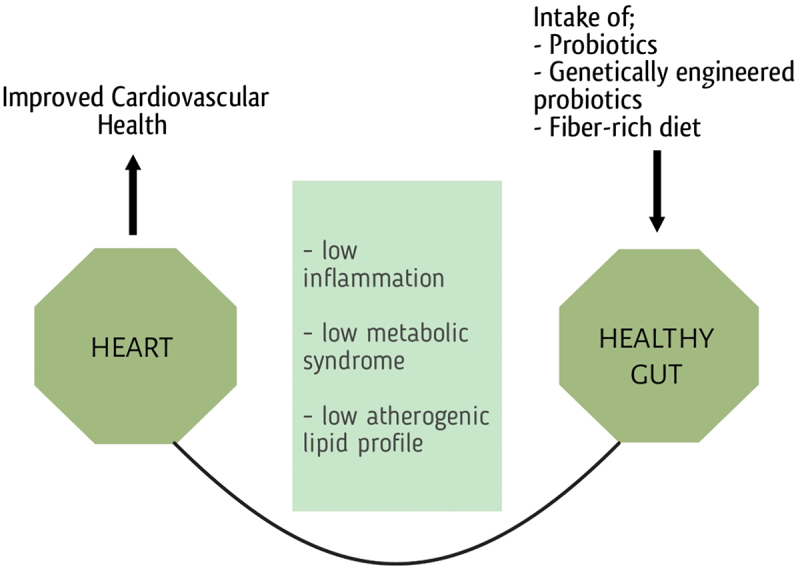
Cardiovascular health is linked with the diet which shapes the microbiota of the gut and leads to a healthy life cycle.

Studies on animal models and human observations suggest diets high in plant-based polysaccharides, fiber, and starch contribute to greater gut microbiota diversity and health benefits, while diets high in fat, sugar, and animal protein contribute to lower diversity and increased risk of obesity and cardiometabolic diseases.^48^ The production of SCFAs plays an important role in modulating the cardiometabolic risk associated with diet. Diets rich in fiber or supplemented with prebiotic fibres have a favorable effect on gut microbiota composition, while high-fat diets, particularly those high in saturated fatty acids, have the opposite effect.^103^ Figure 8 shows the intake of a particular diet and its relation to heart health.

Several bacteria present in the gut are responsible for cardiometabolic diseases and are discussed in Table 3 given below.

**Table 3.** Association of gut flora with various diseases.

Disease	Gut Microbes	Findings
Cardiovascular diseases	*Lactobacillus plantarum* *Lactobacillus rhamnosus* *Akkermansia muciniphila*	Improved ventricular function Improved blood pressure Protect against atherosclerosis
Type 2 Diabetes	*Bifidobacterium*, *Bacteroides*, *Faecalibacterium, Akkermansia and Roseburia spp.*	Negatively associated
	*Ruminococcus*, *Fusobacterium*	Positively associated
	*Lactobacillus* species	Enriched in control microbiome.

#### 1.6.2. Aging

**Figure 9.**
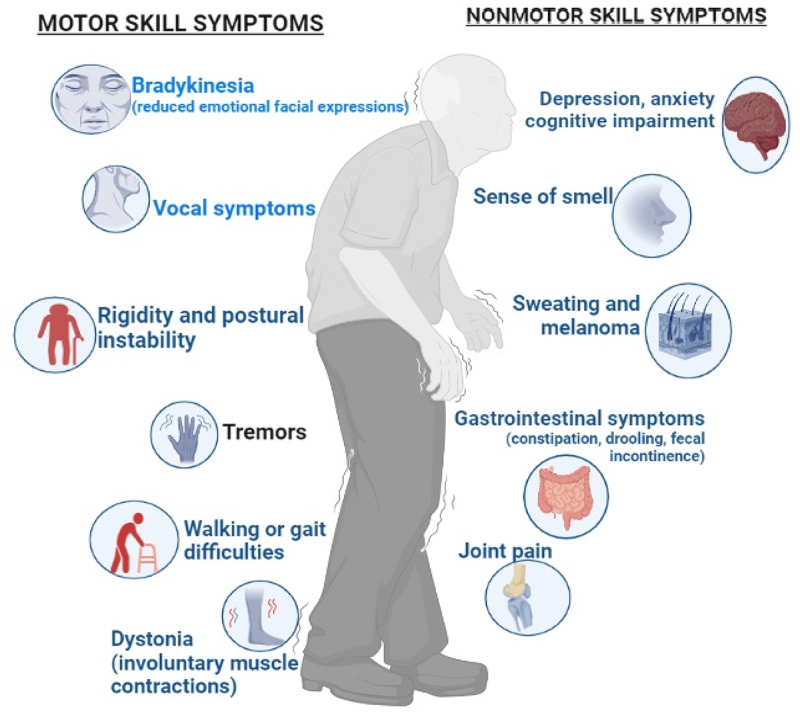
Symptoms of aging with gut diseases.

Aging is a complex biological process characterized by a progressive decline in the functional capacities of an organism over time. Several motor as well as non-motor symptoms are there which represent the aging process (Figure 9). Numerous factors contribute to aging, including both genetic and environmental influences in which diet is substantial. A healthy and balanced diet, rich in nutrient-dense foods, can help prevent age-related diseases, support cellular repair mechanisms, and promote overall well-being. Following are some of the symptoms discussed from both the motor and non-motor skills category.

##### 1.6.2.1. Motor Skills Symptoms

###### 1.6.2.1.1. Impaired Coordination

The nervous system can be affected by some gut diseases, such as celiac disease, resulting in impaired coordination and balance. Consequently, individuals may experience difficulties with motor skills, including walking, fine motor tasks, or hand-eye coordination.

###### 1.6.2.1.2. Muscle Weakness

Inflammatory myopathies or nutritional deficiencies due to malabsorption, caused by certain gut diseases, can lead to muscle weakness in older adults. Such weakness can result in difficulties with mobility, standing, and performing physical tasks that require strength.

###### 1.6.2.1.3. Tremors

Tremors or involuntary muscle movements can be associated with gut diseases in some cases. These tremors may impair fine motor abilities, making it difficult to write or button clothing.^104^

###### 1.6.2.1.4. Motor Impairments

Motor deficits may occur in older persons with gastrointestinal conditions that impact the neurological system, such as Parkinson’s disease or multiple sclerosis. These impairments may present as difficulties with balance, gait disturbances, muscle stiffness, and slowed movements .^105^

##### 1.6.2.2. Non-motor skills symptoms

###### 1.6.2.2.1. Cognitive Impairment

Gut diseases have been linked to cognitive symptoms, including brain fog, memory problems, difficulty concentrating, and mental fatigue. In older adults, these symptoms may be more noticeable and impact daily functioning.^106^

###### 1.6.2.2.2. Mood Disorders

Mood disorders, such as depression and anxiety, can be attributed to gut diseases since gut health is closely tied to mental health. Older adults with gut diseases may experience changes in mood, emotional instability, or a decreased sense of well-being.

###### 1.6.2.2.3. Fatigue and Weakness

Chronic gut diseases can cause fatigue and weakness, which can impact both physical and cognitive abilities. Older adults may experience increased fatigue, reduced stamina, and a decreased ability to engage in daily activities.^107^

###### 1.6.2.2.4. Sleep Disturbances

Gut conditions can interfere with sleep cycles by making it difficult to fall asleep, stay asleep, or get enough rest. Sleep disturbances can further contribute to fatigue, cognitive impairment, and mood disorders).^108^

###### 1.6.2.2.5. Nutritional Deficiencies

Nutrient absorption can be impaired by gut diseases, leading to nutritional deficiencies. Inadequate nutrition can affect overall energy levels, cognitive function, and overall health, which may manifest as non-motor symptoms in older adults.^44^

#### 1.6.3. Polymyalgia rheumatica (PMR)

PMR is an inflammatory illness that primarily impacts people over 50 years old. C-reactive protein (CRP, as well as erythrocyte sedimentation rate (ESR),) are both at high levels in them. Even though the precise cause of the PMR is not known, there exists proof that indicates that eating habits contribute to the onset and progression of the condition. According to several research, people with PMR may benefit from taking dietary supplements like vitamin D. For instance, a 2015 study discovered that vitamin D supplementation may help PMR patients’ symptoms and lessen their need for corticosteroid therapy.^109^ According to a 2019 study,^110^ consuming processed foods, red and processed meat, and refined sugars may increase the risk of PMR. However, a diet high in fruits, vegetables, sources of protein, and whole grains may help avoid PMR. Ruminococcus, Prevotella, and Bacteroides were much more prevalent in the stomachs of those with the illness compared to the microbial community of the gut in healthy normal individuals, suggesting that these bacterial species may be linked to this ailment.^46^ A balanced diet consisting of fruits, whole grains, a range of veggies, legumes, seeds, and nuts will enhance the consumption of prebiotic fibres that nourish the healthy bacteria in the gut. Fermented foods like yogurt are helpful for probiotic bacteria because they promote gut flora. In addition, limit the consumption of processed foods, saturated and trans fats, and sugars since these can be a major factor in inflammation.^111^ However, rather than healing inflammatory diseases, all of the aforementioned foods can reduce their symptoms.

#### 1.6.4. Spinal muscular atrophy (SMA)

In the treatment of SMA patients, nutrition plays a big role. These patients experience increasing muscular atrophy, and the functional limitations they experience have a disastrous impact on the results of diet.^112^ A unique diet and dietary fiber consumption are essential to preserve the excellent health of this particular disease’s patients because so many of them struggle with malnutrition.^113^ In Boston, Massachusetts, a study was conducted. After following a particular diet and diet for three years among the 60 participants who participated in the trial, the prevalence of malnutrition fell from 65% to 27%.^114^ 36 studies were reviewed comprehensively in Australia. All of this research has demonstrated how crucial dietary fibres are to SMA patients’ ability to grow normally and strengthen their muscles.^63^ In 2016, a different research on the Chinese populace was conducted. It revealed that 84% of the study’s participants had poorer calcium absorption rates and were undernourished. They concluded that a particular diet and calcium intake could enhance the growth of the body and muscles.^115^ According to studies, dietary alterations in the microbiota of the gut are linked with SMA. Another study in which mice were used as animal models for understanding SMA found that the severity and progression of the disease were related to changes in the microbial ecology of the stomach. Comparing SMA mice to control mice, there was a shift in the abundance of various bacterial taxa. The researchers discovered that probiotics were effective in improving motor function and lengthening longevity in SMA-affected mice.^116^ Another study examined the gut flora of some SMA patients to evaluate their health. The researchers discovered that the microbial diversity of these patients was distinct from those of healthy controls. These changes in the microbial ecology of the stomach, according to the scientists, may be important in the development of SMA. Additionally, concentrating on the microbes in the gut might be a cutting-edge way to treat the disease.^117^

#### 1.6.5. Vasculitis

Patients who have vasculitis receive steroids, which might cause osteoporosis. Increased calcium intake can help to stop it from happening. Additionally, including foods like broccoli, skim milk, yogurt, etc. in your diet is advised.^118^ In patients with vasculitis, the gastrointestinal tract involvement is extremely typical. Patients with chronic systemic inflammation typically lose weight and develop anorexia; as a result, nutrition should be adjusted as necessary.^119^ Two groups of mice were used in a different experiment based on their dietary variations. Beta-glucan is absent in one group while all other nutrients are present in the other group. Comparatively, group two, which did not include beta-glucan, had shorter survival times. Additionally, the second group’s gut microbial ecology contained a significant amount of the Bacteroides that promote inflammation.^120^ Researchers have discovered that the levels of antioxidants are inversely associated with the levels of CRP and other inflammatory indicators in various cross-sectional studies. A study group was created with a limited diet that excluded fruits and vegetables and included simple carbs and fried meals. Their findings indicated elevated CRP, which helped to explain the connection between nutrition and disease. Anti-neutrophil cytoplasmic antibody-associated vasculitis, often known as AAV, is one of the kinds of vasculitis in which the patient experiences inflammation of the small blood vessels. The primary players in the development of disease are neutrophils, and the metabolites that are created when the microbial flora of the gut ferment non-digestible carbohydrates have a significant impact on their activity.^121^

#### 1.6.6. Sarcopenia

According to the information at hand, diet may be significantly involved in the development and progression of sarcopenia. It has been shown that older persons need adequate protein intake, especially from high-quality sources, to maintain their muscle mass and function. Vitamin D and omega-3 fatty acids may also be able to stop sarcopenia. For instance, a thorough review and meta-analysis of 20 studies on sarcopenia in older persons found that protein supplementation improved the maintenance of muscular mass, strength, and function.^15^ Another study found that elderly women who had an omega-3-rich diet had higher muscle mass and less inflammation. Finally, a randomized controlled preliminary research concluded that vitamin D administration helped older persons with sarcopenia enhance their muscle strength and capabilities.^58^ Additionally, a high-quality protein source was found to be effective in improving muscular strength, mass, function, and physical activity in persons with sarcopenia who are older than 60.^122^ Furthermore, a cross-sectional study discovered that older persons with higher fruit and vegetable intake had more muscular mass and strength.^123^ Additionally, a randomized controlled trial found that sarcopenic older people’s strength of muscles and physical function was enhanced by a Mediterranean-style diet that is high in veggies, fruit, whole-grain foods, and healthy fats.^124^ Overall, these studies indicate that the prevention and treatment of sarcopenia in older persons can be aided by a balanced diet that includes enough protein, fruits, and vegetables, as well as a Mediterranean-style diet.^125^

#### 1.6.7. Cirrhosis

Instead of healthy liver tissue, cirrhosis, a chronic liver condition, is distinguished by the growth of scar tissue. Diet has a major impact on the onset and progression of cirrhosis, according to a study. One cirrhosis complication that affects brain function is hepatic encephalopathy, and consuming a lot of proteins has been linked to an increased chance of acquiring it.^126,126^ Contrarily, a larger consumption of saturated and monounsaturated fats has been connected to an increased risk of liver fibrosis, whereas an increased consumption of polyunsaturated fatty acids has been associated with a lower risk of liver fibrosis, which is a substantial contributor to cirrhosis. Additionally, cirrhosis, a leading cause of non-alcoholic fatty liver condition (NAFLD), has been linked to a diet high in sugars and refined carbohydrates.^56^ Decreased dietary fiber intake has also been linked to cirrhosis and its side effects, such as liver encephalopathy and variceal hemorrhage, according to studies.^127^ A 2019 study discovered a link between a diet containing a lot of red and processed meat and the risk of fibrosis of the liver, which can lead to cirrhosis.^128^ People with cirrhosis may benefit from a Mediterranean-style diet rich in vegetables, whole grains, fruits, seafood, and olive oil since it improves liver function and reduces the risk of abnormalities.^23^ These findings suggest that dietary modifications may be essential for both cirrhosis prevention and treatment.

#### 1.6.8. Undefined

Studies have suggested a connection between diet and cancer. A 2015 study found a higher risk of colon cancer among people who consume a Western-style diet heavy in processed and red meats, refined cereals, and sugary drinks.^129^ A different 2018 study found that a plant-based diet rich in fruits, vegetables, whole grains, and legumes may help reduce the risk of breast cancer.^130^ Additional research suggests that several nutrients, including omega-3 fatty acids and Vit-D, may also act as a preventative step against a variety of cancers.^29,131^

#### 1.6.9. Fibromyalgia

Diet and nutrition are two areas of a person’s life that are impacted by the complex chronic pain syndrome known as fibromyalgia. According to numerous research, dietary components may have an impact on the development and management of fibromyalgia. For instance, a 2017 study revealed that a low-FODMAP diet, which limits particular carb types that may cause issues with digestion, may be beneficial in easing fibromyalgia symptoms. Further research has shown that a diet rich in foods that are anti-inflammatory, such as produce, whole grain products, and lean protein, may benefit those with fibromyalgia.^14,132^

#### 1.6.10. Alzheimer’s disease

Memory loss and dementia are hallmarks of the degenerative neurological disorder Alzheimer’s disease. Even though the exact cause of Alzheimer’s disease is unidentified, study evidence suggests that diet and gut flora may have an impact on the onset and course of the illness. One study found that the gut microbiota of people with Alzheimer’s disease was different from that of healthy people, with lower numbers of beneficial bacteria and higher levels of potentially harmful bacteria.^133^ In another study,^134^ it was discovered that changes in the gut microbiota may enhance oxidative stress and inflammation, both of which have been associated with the development of Alzheimer’s disease. According to studies on nutrition, a Mediterranean-style diet has been associated with a lower incidence of Alzheimer’s disease.^135^ Whole grains, fresh produce, fruits, and healthy fats are prioritized in this diet. Many of the foods in this type of diet are thought to contain anti-inflammatory and antioxidant properties, which may be the reason for this. A diet high in sugar, processed carbohydrates, and trans fats, on the other hand, has been connected to an increased risk of Alzheimer’s disease.^22^ Even though the connection between Alzheimer’s disease, gut microbial flora, and food is still being researched, there is evidence to suggest that a nutrient-rich diet and a balanced microbial flora of the gut may be important factors in lowering the risk of getting the disease.

#### 1.6.11. Parkinson’s disease

Parkinson’s disease is a disease of the nervous system that is characterized by the death of dopamine-producing neurons in the brain. According to recent studies, food and gut flora may affect how the sickness manifests and worsens. According to studies, patients suffering from this disease have a different gut microbiota than people in good health, with less beneficial bacteria and a higher concentration of potentially harmful bacteria.^136^ Elevated inflammatory conditions, which have been associated with changes in the intestinal microbiota in earlier studies, are thought to accelerate the progression of Parkinson’s disease.^137^ Food research has shown that a high-fiber diet is associated with a decreased risk of Parkinson’s disease.^138^ The development of good gut flora is thought to be supported by fiber, and this may assist in minimizing oxidative stress and inflammation. Conversely, a diet high in processed foods and saturated fats has been linked to an increased incidence of Parkinson’s disease.^139^ Fiber may help to reduce inflammation and oxidative stress by supporting the growth of healthy gut flora.

#### 1.6.12. Lupus

Systemic lupus erythematosus (SLE), another name for lupus, is an autoimmune disorder that can damage many body organs.^93^ Research suggests that diet and the bacteria in the gut may affect the progression and course of the disease. Another study found that persons with SLE had a different makeup of the bacteria in their stomachs than people in the general population, with less good bacteria and more potentially harmful bacterial species.^140^ Other studies suggest that changed gut microbiota may contribute to an increase in inflammation, a key factor in the development of SLE.^141^ A Mediterranean-style diet was found to be linked with a lower risk of developing SLE in a food research study^142,94^ This is thought to be the case since many of the foods in this type of diet have antioxidant and anti-inflammatory properties. However, a diet abundant in processed foods, sweets, and saturated fats is strongly linked to an increased risk of SLE.^143^ It is hypothesized that a good diet and a healthy gut microbiota may be significant factors in lowering the risk of getting lupus and controlling its symptoms, although research into the relationship between lupus, the microbiota of the gut, and food is currently ongoing.

#### 1.6.13. Arthritis

Arthritis is a term that is frequently used to describe joint pain and inflammation. According to research, certain types of arthritis are more likely to develop and progress if particular foods and gut flora are consumed. According to research, patients with rheumatoid arthritis (RA) have a different gut microbiota than healthy individuals, with less beneficial bacteria and a higher concentration of potentially harmful bacteria.^144^ Inflammation, which has been connected in other studies to changes in the gut microbiota, is primarily responsible for the development of RA.^145^ According to a dietary study, a Mediterranean-style diet lowers the chance of developing RA.^146^ This is thought to be the case since many of the foods in this type of diet have antioxidant and anti-inflammatory properties. On the other hand, Chen et al.^147^ found a relationship between a diet high in processed foods, saturated fats, and sugar with an increased chance of developing RA.^147^ Additionally, it has been demonstrated that a variety of foods possess anti-inflammatory qualities, which may help treat the symptoms and signs of arthritis. As an illustration, omega-3 fatty acids, which are found in fish oil, have been demonstrated to lower inflammation and may benefit people with arthritis by reducing joint discomfort and rigidity.^148^ Even though the connection between arthritis, food, and gut microbiota is still being studied, there is evidence to suggest that a healthy diet and a balanced gut microbiota may be important factors in reducing the likelihood of getting the disease and controlling its symptoms.

#### 1.6.14. Constipation

As individuals age, they may experience changes in their digestive system, including decreased muscle tone and slower transit time in the colon. These age-related changes can contribute to a higher prevalence of constipation among older adults.^149^ Studies suggest that alterations in the gut microbiota composition and diversity may play a role in the development of constipation. Imbalances in the gut microbiota, characterized by a reduction in beneficial bacteria and an overgrowth of potentially harmful bacteria, have been associated with constipation.^150,151^ Diet is a major factor influencing the composition and activity of the gut microbiota. A diet rich in fiber, prebiotics (e.g., inulin, fructooligosaccharides), and resistant starch can promote the growth of beneficial bacteria in the gut and enhance bowel movements.^152,153^ On the other hand, a low-fiber, highly processed diet may negatively affect the gut microbiota diversity and function, potentially contributing to constipation. Research suggests that dietary factors influence constipation through their effects on the gut microbiota. For example, a study found that a high-fiber diet increased stool frequency and improved constipation symptoms through the promotion of the growth of beneficial bacteria, including *Bifidobacterium* and *Lactobacillus*, in the gut. Conversely, diets low in fiber and high in fat and sugar have been associated with an increased risk of constipation and alterations in the gut microbiota.^154^

### 1.7. Gut health can reduce the risk of these diseases

**Figure 10.**
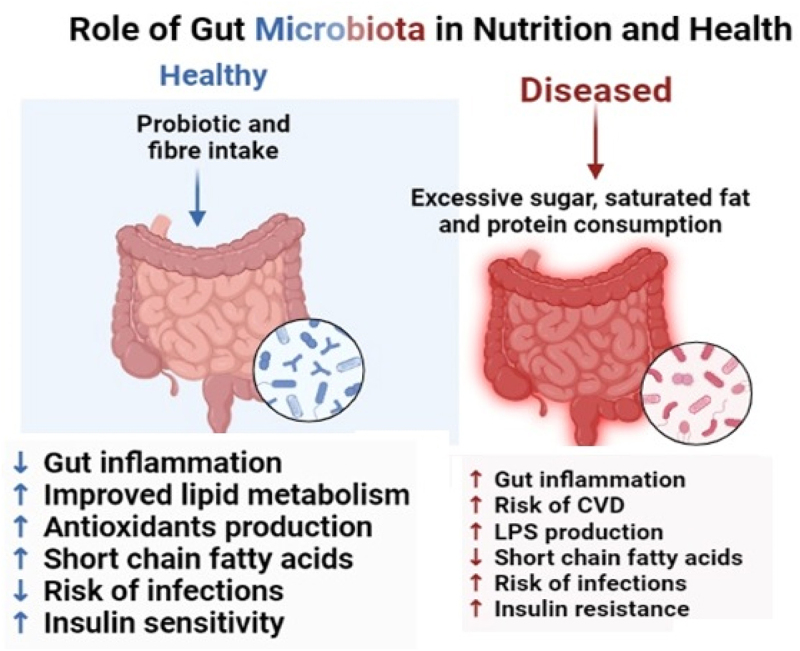
Gut health reduces the risk of various diseases.

The microbiota of the gut, which is made up of hundreds of millions of bacteria living in the gastrointestinal tract, has a substantial impact on the functioning of one’s immune system and the body’s immunological response. According to recent studies, the composition and functioning of the gut microbiota can be significantly influenced by dietary factors as well as other factors. The body’s ability to fight off diseases and infections and immunological function are both significantly influenced by the gut microbiota.^155^ A nourishing, balanced diet that is low in inflammatory foods can strengthen an immune system. Genetics and lifestyle both play a significant role in the relationship between nutrition and immune health, making it challenging. Two eating patterns that are well-liked among Europeans are the Western diet and the Mediterranean diet. The Mediterranean diet, which prioritizes whole grains, fruits, and vegetables as well as healthy fats, has been linked to reduced levels of inflammation and a lower chance of developing chronic diseases including cancer and cardiovascular disease. The Western diet, however, has been associated with elevated inflammation and a higher risk of chronic illnesses since it is high in processed foods, red and processed meat, and refined carbohydrates. Western diets that are frequently high in saturated and trans fats may promote inflammation and impair immune system function. Consuming excessive amounts of sugar can also lead to chronic inflammation and raise the risk of autoimmune diseases.^2^ Eating patterns may affect how well the immune system functions. Intermittent fasting, which involves restricting food intake for a predetermined amount of time, has been shown in animal studies to improve immunological function. People’s immune function is improved by intermittent fasting and to have infy markers reduced.^3^ Inflammatory diseases including Inflammatory Bowel Disease (IBD) have been linked to unbalanced gut flora. A growing amount of evidence suggests that nutrition has a significant influence on the onset and course of many illnesses by changing the composition and efficiency of the gut microbial flora.^156^ According to studies, diets high in refined carbohydrates and saturated fats may decrease the number of beneficial bacteria in the stomach while increasing the number of potentially dangerous bacteria. In addition, diets higher in fiber, vegetables, fruits, and whole grains have been found to promote the growth of beneficial bacteria and reduce gastrointestinal inflammation (Figure 10).^107^

Two dietary components, prebiotics and probiotics, have regulatory effects on people with inflammatory diseases. Prebiotics are non-digestible fibres that encourage the growth and activity of beneficial bacteria in the stomach. When consumed in the right amounts, probiotics can improve health by introducing beneficial live bacteria into the digestive system.^157^ In general, food can have a substantial impact on a person’s stomach’s microbial flora if they have an inflammatory sickness. By promoting a healthy and diverse gut microbiota through dietary therapy, it may be possible to shorten the course of some illnesses and diminish symptoms. By changing the diet to foster the optimum bacterial habitat in the gut, it is feasible to manage the symptoms and signs of these diseases as well as minimize their effects.^153^ Table 4 summarizes some of the disorders mentioned above and lists the foods to stay away from to treat them.

**Table 4.** Summary of the association between inflammatory diseases and gut microbiota concerning diet.

Common Inflammatory Diseases	Beneficial Diet	Harmful Diet	Changes in Gut Microbial flora due to harmful diet	References
PMR	Vitamin D, fruits, vegetables, whole grains, lean proteins	Processed foods, refined sugars, fats	Increase in *Prevotella*, *Bacteroides*, *Ruminococcus* sp.	^109^ ^110^
SMA	Calcium, dietary fibers, probiotics	Processed foods	Increase in harmful bacteria.	^115^ ^116^
Vasculitis	Calcium, broccoli, yogurt, skimmed milk, fruits, vegetables, beta-glucan	Food additives, fried foods, non-digestible carbohydrates	Increase in *bacteroides*	^118^ ^120^ ^121^
Sarcopenia	Proteins, vit.D, omega 3 fatty acids, fruits, vegetables	Western style foods including fats and processed sugars and foods	Increase in harmful bacteria.	^158^ ^58^ ^124^
Cirrhosis	Polyunsaturated fats, dietary fibres, whole grains, omega-3 foods (fish, olive oil)	Saturated and monounsaturated fats, high intake of processed and red meat, sugars and refined carbohydrates	Increase in harmful gut bacteria	^159^ ^56^ ^23^
Cancer	Plant-based diet; fruits, vegetables, legumes, whole grains, vitamin D, omega 3 fatty acids	High intake of processed and red meat	Increase in inflammation causing bacteria	^129^ ^130^ ^29,131^
Fibromyalgia	Low FODMAP diet, fruits, vegetables, lean proteins, whole grains	Processed foods and carbohydrates	Increase in Inflammation causing flora	^160^ ^14; 132^
Alzheimer’s	Mediterranean diet	Western diet	Inflammation and oxidative stress	^135^
Parkinson Disease	High fiber diet	Saturated fats and processed foods	Inflammation and oxidative stress	^139^
Lupus	Mediterranean diet	Western diet	Change in gut microbiota and inflammation	^143^
Arthritis	Mediterranean diet	Processed foods, sugars, saturated fats	Inflammation and oxidative stress	^147^
Constipation	Fiber, prebiotics	Low fiber diet, fats, sugars	Change in microbial flora of gut	^154^

### 1.8. Challenges to overcome this problem

There are several challenges that researchers and individuals face when it comes to understanding and harnessing the relationship between diet and the gut microbiota. Some of these challenges include:

1. *Inter Inter-individuality*: Each person has a unique gut microbiota composition, which can vary significantly between individuals. This inter-individual variability makes it challenging to establish universal dietary recommendations that can effectively modulate the gut microbiota for everyone. Personalized approaches and more extensive research are needed to understand the impact of specific dietary interventions on diverse populations.
2. *Gut Microbiota’s Complexity*: The microbial flora of the gut is an incredibly complex environment having millions and trillions of microbes with diverse species and strains. Understanding the interactions and functions of these microorganisms and their response to different dietary components is a complex task. The vastness and intricacy of the gut microbiota present challenges in comprehensively mapping its composition and functionalities.
3. *Limited Understanding of Mechanisms*: While research has made significant strides in understanding the role of gut microbiota in human health, many mechanisms underlying the diet-microbiota interaction remain poorly understood. Scientists are still unravelling how specific dietary components influence the gut microbiota, and how these microbial changes translate into health outcomes. Further research is needed to elucidate these mechanisms.
4. *Lack of Long-term Studies*: Most studies investigating the diet-microbiota relationship are short-term and often limited to specific populations or interventions. Long-term studies are necessary to assess the stability and long-lasting effects of dietary interventions on the gut microbiota. Additionally, conducting controlled trials over extended periods can be challenging and resource-intensive.
5. *Standardization of Research Methods*: There is a lack of standardized research methods and techniques for studying the gut microbiota. Variations in sampling, sequencing methods, and data analysis can make it difficult to compare and replicate study findings. Developing standardized protocols and approaches will help ensure consistency and reliability in gut microbiota research.
6. Translation into Practical Recommendations: While there is growing scientific knowledge about the diet-microbiota relationship, translating this knowledge into practical dietary recommendations for individuals can be challenging. Factors such as individual differences, cultural preferences, and dietary habits need to be considered when developing personalized dietary guidelines based on gut microbiota research.
7. *Ethical Considerations*: As researchers explore potential interventions that can modulate the gut microbiota, ethical considerations arise. It is crucial to ensure the safety, informed consent, and equitable access to any interventions or therapies targeting the gut microbiota. Ethical guidelines and careful oversight are necessary to protect the well-being and autonomy of study participants.

Addressing these challenges will contribute to a better understanding of how dietary interventions can be optimized to promote gut health and overall well-being.

### 1.9. Current trends

Some of the current trends include personalized nutrition. Researchers are exploring the concept of personalized nutrition, which takes into account an individual’s unique gut microbiota composition and dietary needs. This approach aims to develop personalized dietary recommendations to optimize gut health and overall well-being. Second comes high-fiber diet. There is growing recognition of the importance of dietary fiber for gut health. High-fiber diets, including diverse plant-based foods, are associated with a more diverse and beneficial gut microbiota. Such diets are linked to various health benefits, including improved digestion, reduced inflammation, and better metabolic health. The third one is a plant-based diet. Plant-based diets, such as vegetarian or vegan diets, have gained popularity. These diets typically emphasize whole plant foods and are often rich in fiber, prebiotics, and phytonutrients. Plant-based diets have been associated with positive effects on the gut microbiota, including increased levels of beneficial bacteria and microbial diversity.^161^ Fourth are the fermented Foods. Fermented foods, such as yogurt, kefir, sauerkraut, kimchi, and kombucha, have gained attention due to their potential probiotic benefits. These foods contain live beneficial bacteria that can support gut health by enhancing microbial diversity and function. Fifth are the prebiotics and probiotics. Prebiotics are indigestible fibres that serve as food for beneficial gut bacteria, while probiotics are live bacteria that can confer health benefits when consumed. There is ongoing research into the specific probiotic strains that have targeted impacts on gut health and overall well-being. The Gut-Brain Axis is the sixth one and refers to the bidirectional communication between the gut and the brain. Emerging research suggests that the gut microbiota plays a crucial role in this communication and can affect the functioning of the brain, mental health, and overall mood of the individual. Scientists are studying the potential of modulating the gut microbiota through diet to improve mental well-being. Lastly, postbiotics which are bioactive compounds produced by beneficial gut bacteria during fermentation are included in this list. They can have direct beneficial effects on the host. Research is exploring the potential health benefits of postbiotics and their role in modulating gut microbiota and overall health.^162–170^

### 1.10. Recommendations

As people age, it becomes increasingly important to pay attention to their diet and the health of their gut microbiota. Following are some recommendations for aged individuals:

1. Eat a varied and balanced diet: Encourage a diet rich in fruits, vegetables, whole grains, lean proteins, and healthy fats. A diverse range of foods helps support a diverse gut microbiota.
2. Increase fibre intake: Include plenty of fibre-rich foods such as whole grains, legumes, fruits, and vegetables in the diet. Fiber promotes regular bowel movements, supports gut health, and feeds beneficial gut bacteria.
3. Consume probiotic and prebiotic foods: Probiotics are live bacteria that can have beneficial effects on gut health. Yogurt, kefir, sauerkraut, kimchi, and other fermented foods contain natural probiotics. Prebiotic foods, like garlic, onions, bananas, and asparagus, provide nourishment for the beneficial gut bacteria.
4. Stay hydrated: Drinking an adequate amount of water is essential for maintaining optimal digestive function and preventing constipation. Aim for at least 8 cups (64 ounces) of water per day, or more if needed.
5. Limit processed foods and added sugars: Highly processed foods and excessive added sugars can negatively impact gut health. They can disrupt the balance of gut bacteria and contribute to inflammation. Encourage whole, minimally processed foods instead.
6. Consider a multivitamin or supplement: Aging individuals may have specific nutrient needs. Consult a healthcare professional to determine if a multivitamin or specific supplements, such as vitamin D or omega-3 fatty acids, are necessary.
7. Avoid unnecessary antibiotic use: Antibiotics can disrupt the balance of gut bacteria. While sometimes necessary, it’s important to avoid unnecessary or excessive antibiotic use to maintain a healthy gut microbiota.
8. Manage stress: Chronic stress can impact gut health. Encourage stress management techniques like exercise, meditation, or hobbies to support overall well-being, including gut health.
9. Stay physically active: Regular exercise supports healthy digestion and can positively influence the gut microbiota. Encourage aging individuals to engage in activities they enjoy and maintain a physically active lifestyle.
